# The Formation of 2,2,4-Trimethyl-2,3-dihydro-1*H*-1,5-Benzodiazepine from 1,2-Diaminobenzene in the Presence of Acetone

**DOI:** 10.3390/molecules181114293

**Published:** 2013-11-19

**Authors:** Felix Odame, Phumelele Kleyi, Eric Hosten, Richard Betz, Kevin Lobb, Zenixole Tshentu

**Affiliations:** 1 Department of Chemistry, Nelson Mandela Metropolitan University, P.O. Box 77000, Port Elizabeth 6031, South Africa; E-Mails: felixessah@yahoo.com (F.O.); pkleyi@yahoo.com (P.K.); eric.hosten@nmmu.ac.za (E.H.); richard.betz@nmmu.ac.za (R.B.); 2 Department of Chemistry, Rhodes University, P.O. Box 94, Grahamstown 6140, South Africa; E-Mail: k.lobb@ru.ac.za

**Keywords:** *o*-phenylenediamine, acetone, benzodiazepine

## Abstract

In an attempt to synthesize a 2-substituted benzimidazole from the reaction of *o*-phenylenediamine and isophthalic acid in the presence of acetone and ethanol under microwave irradiation, a salt of the isophthalate ion and 2,2,4-trimethyl-2,3-dihydro-1*H*-1,5-benzodiazepin-5-ium ion was obtained. The condensation of two moles of acetone with the amine groups resulted in the formation of the benzodiazepine which crystallized as an iminium cation forming a salt with the isophthalate anion. The formation of benzodiazepine was also confirmed by performing the reaction of *o*-phenylenediamine with excess acetone in ethanol under conventional heating conditions. The compounds were characterized by NMR, FTIR, HRMS and microanalysis as well as X-ray crystallography. The reaction mechanism leading to the formation of benzodiazepine is also discussed.

## 1. Introduction

Reactions of *o*-phenylenediamine with a dicarboxylic acid can produce several different products depending on the specific conditions [1]. In the presence of cyclization agents such as hydrochloric acid or polyphosphoric acid, these reactions have been reported to give benzimidazoles [2,3]. This is a condensation reaction that is initiated by the protonation of the carbonyl group oxygen. Subsequently, the attack of the carbonyl carbon by the lone pair of electrons on the amino group results in the formation of benzimidazole with the loss of two molecules of water [4]. Another possible product is dibenzimidazole, which is a result of the cyclization or condensation of both carboxylic acids groups with the diamine [5]. This reaction easily happens when two moles of *o*-phenylenediamine are reacted with the dicarboxylic acid in the presence of polyphosphoric acid in xylene [6]. Amides (monoamides, diamides or polyamides) could also be formed by the reaction between an acid and an amine in the presence of a mineral acid under reflux [7]. There is also the possibility of a reaction between two dicarboxylic acid molecules to form an anhydride with the loss of a molecule of water.

The synthesis of diazepines via various synthetic procedures under catalyzed conditions has widely been reported [8,9,10,11,12,13]. Phenolic β-diketones have been converted to flavones in acidic medium which on treatment with aqueous ethylenediamine or propylenediamine gave diazepine derivatives [8]. 1,2-Diazepine derivatives have been synthesized by the reaction of (1*Z*)-1-[(2*E*)-3-(4-bromophenyl)-1-(4-fluorophenyl)prop-2-ene-1-ylidene]-2-(2,4-dinitrophenylhydrazine with chloroacetate in the presence of a base [9]. Kaoua and co-workers have reported the synthesis of diazepines by the reaction of ketimine intermediates and aldehydes in the presence of Keggin-Type heteropolyacids (HPA) [10]. The nucleophilic substitution of coumarincarbaldehyde derivatives with diamines resulted in the formation of 1,4-benzenediazepines [11]. Rekha *et al.* have reported similar benzodiazepine derivatives by the condensation of *o*-phenylenediamine and a ketone or an aldehyde in the presence of a catalyst (alumina and zirconia) [12]. The formation of 7-membered ring diazepine systems by microwave irradiation of a mixture of an aldehyde, a ketone and ethylenediamine in the presence of potassium hydroxide has also been reported [12,13]. Diazepine-like complexes have been synthesized in an acetone medium where the metal centre forms part of the six-membered ring [14,15,16,17]. The metal precursors included Ni(II) [14], Ir(I) and Ir(III) [15], Rh(III) [16] and Co(III) [17]. In most cases the six-membered ring was formed via the condensation of the ammine ligand coordinated to the metal centre and the acetone [14,15,17]. In another study, the six-membered ring formed via the aldol-type condensation of two acetimino ligands coordinated to the metal centre [16].

This communication reports on the formation of benzodiazepine from the reaction of *o*-phenylene-diamine and isophthalic acid under microwave irradiation in the presence of a acetone-ethanol mixture, and in the absence of a catalyst. The formation of this product was also confirmed by a reaction of *o*-phenylenediamine with acetone in ethanol under conventional heating conditions to yield the benzodiazepine.

## 2. Results and Discussion

### 2.1. Chemistry

**Scheme 1:**
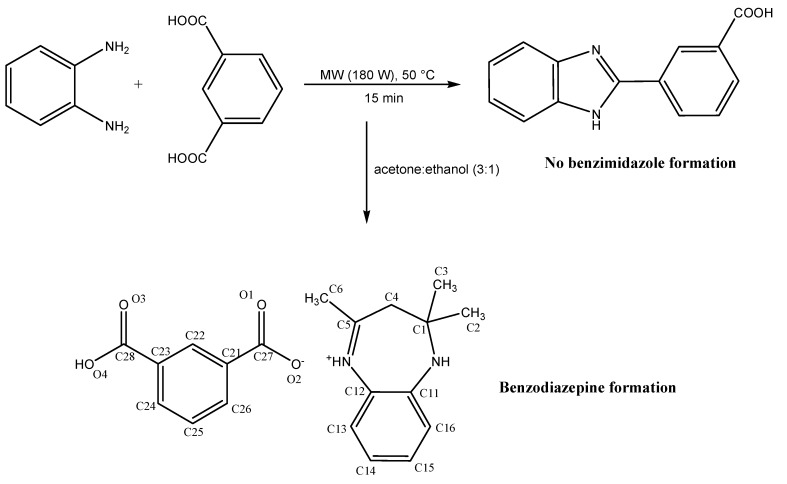
Synthesis of 2,2,4-trimethyl-2,3-dihydro-1*H*-benzodiazepin-5-ium isophthalate (**I**).

The initial objective was to form a benzimidazole from the reaction of *o*-phenylenediamine and isophthalic acid under microwave irradiation conditions. Equimolar quantities (0.01 mol) of the starting materials were irradiated at 180 W (50 °C) for 15 min. To dissolve the solidified reaction mixture, an ethanol-acetone mixture (2 mL, v:v = 3:1) were added while the reaction mixture was still hot. Finally, the reaction mixture was allowed to stand for 12 h. Scheme 1 illustrates the reaction of *o*-phenylenediamine and isophthalic acid under microwave irradiation conditions. 

It was later observed that the benzimidazole formation *via* a condensation reaction had not taken place. This was attributed to the fact that the activation energy required for the reaction of *o*-phenylenediamine and isophthalic acid was not achieved. However, the energy acquired during the microwave irradiation was not immediately lost. This is because there was no other reagent, be it a solid support or solvent to absorb the energy acquired by the reactants during the microwave irradiation. The introduction of an acetone-ethanol mixture into the reaction resulted in the reaction of acetone with *o*-phenylenediamine to yield the benzodiazepine, which was protonated by the isophthalic acid to form a benzodiazepium salt, due to a lower activation energy required for this reaction (Scheme 1). The monocarboxylate anion formed from the dicarboxylic acid resulted in the formation of a salt with the benzodiazepinium cation. Interestingly, it appeared that the reaction occurred without the involvement of isophthalic acid, except in the salt formation. This phenomenon was further ascertained by performing the reaction of *o*-phenylenediamine and acetone under reflux condition in the absence of isophthalic acid.

When 0.02 mol of *o*-phenylenediamine were heated under reflux (80 °C) with ethanol (10 mL) and acetone (5 mL) for 8 h, the product obtained was 2,2,4-trimethyl-2,3-dihydro-1*H*-1,5-benzodiazopine which confirmed that the cyclization occurred without the involvement of isophthalic acid.

The synthesized compounds were characterized using IR and NMR spectroscopy (^1^H, ^13^C), mass spectrometry. as well as elemental analysis. All the characterization data were in agreement with the proposed structures of the compounds. The IR spectrum for 2,2,4-trimethyl-2,3-dihydro-1*H*-1,5-benzodiazepin-5-ium isophthalate (**I**) showed a band at 1,710 cm^−1^ due to the presence of the carbonyl group (C=O) of the carboxylic acid (Supplementary Materials, Figure S1). The band at 1,607 cm^−1^ was attributed to the presence of the iminium group (C=NH+). The bands at 1,208 and 1,552 cm^−1^ indicated the presence of the C–N bond and the C–O bond of the carboxylate ion, respectively. The band at 3,309 cm^−1^ confirmed the presence of the amine group (NH). Furthermore, the IR spectrum of 2,2,4-trimethyl-2,3-dihydro-1*H*-1,5-benzodiazepine (**II**) showed bands at 3,294 cm^−1^ for the amine group (N–H) and a band at 2,964 cm^−1^ for the methyl groups (Supplementary Materials, Figure S2). The bands at 1,633 and 1,430 cm^−1^ were observed for the presence of an imine group (C=N) and a C–N group, respectively.

**Figure 1:**
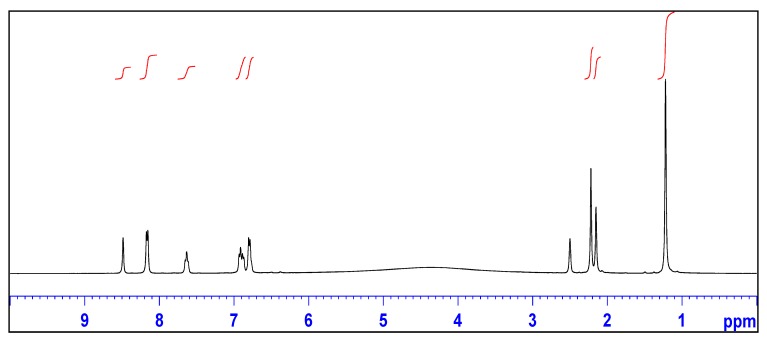
^1^H-NMR spectrum of 2,2,4-trimethyl-2,3-dihydro-1*H*-1,5-benzodiazepin-5-ium isophthalate (**I**).

**Figure 2:**
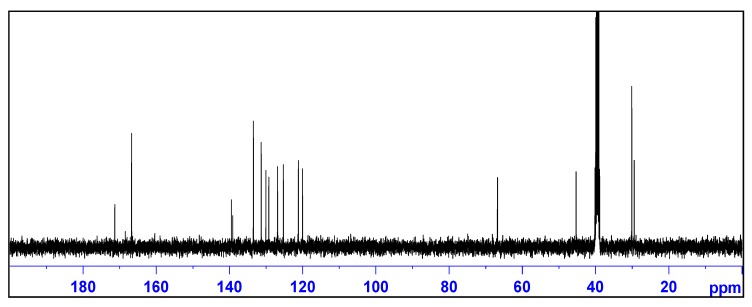
^13^C-NMR spectrum of 2,2,4-trimethyl-2,3-dihydro-1*H*-1,5-benzodiazepin-5-ium isophthalate **I**.

The ^1^H-NMR spectrum of **I** displayed a singlet at *δ* = 2.16 ppm indicating the presence of methylene (CH_2_) protons (Figure 1). The presence of the methylene group was also confirmed by both the ^13^C-NMR spectroscopy (*δ* = 45.24 ppm) (Figure 2) and DEPT-135 (Supplementary Materials, Figure S3. The iminium proton appeared as a broad signal between *δ* = 3.50 and 4.50 ppm. The carbon signal at *δ* = 29.95 ppm was attributable to the two methyl groups attached to the sp^3^ carbon atom of the 7-membered ring. On the other hand, the singlet at *δ* = 29.32 ppm was attributable to a methyl group attached to the sp^2^ carbon of the 7-membered ring. The signals at *δ* = 171.00 and 166.58 were attributable to the carbon atom of the iminium ion and carbonyl groups of the isophthalate anion, respectively.

The ^1^H-NMR spectrum of **II** displayed a singlet at *δ* = 2.16 ppm which integrated for two hydrogens, indicating the presence of CH_2_ group. The presence of the CH_2_ group was also confirmed by the inversion of the corresponding signal (*δ* = 45.23 ppm) in the DEPT-135 spectrum. The N–H group appeared as a singlet at *δ* = 4.71 ppm in the ^1^H spectrum. The singlet at *δ* = 1.24 ppm which integrated for six hydrogens was attributable to the methyl groups attached to the quaternary sp^3^ carbon, whilst the singlet signal at *δ* = 2.22 ppm with integration for three hydrogens was attributable to the methyl group at position 4 on the seven-membered ring.

The main fragments in the high resolution mass spectra (in negative and positive mode) were *m/z* 165.0195 for the isophthalate ion (C_8_H_5_O_4_^−^) and *m/z* 189.1392 for the benzodiazepinium ion (C_12_H_17_N_2_^+^) and these were also consistent with the expected molecular ions peaks of 165 and 189 respectively (Figures S4 and S5, Supplementary Materials).

### 2.2. X-ray Crystallography

**Figure 3:**
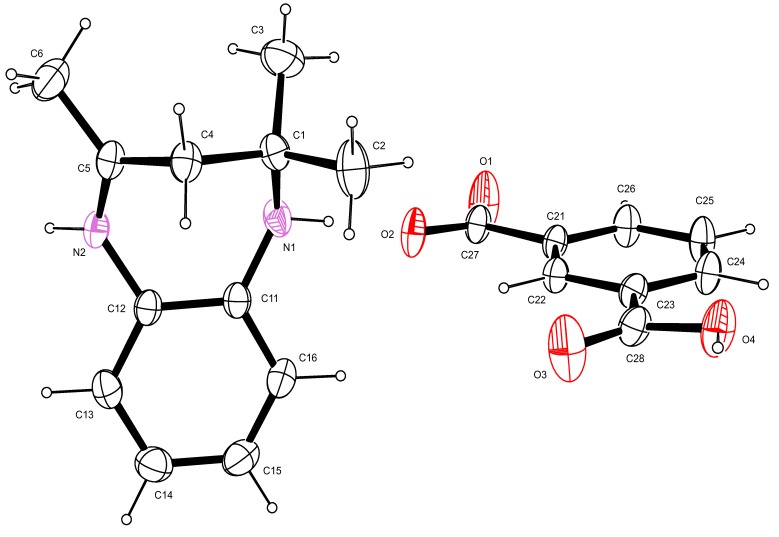
An ORTEP view of I showing 50% probability displacement ellipsoids and the atom labelling.

X-ray crystal structures of the compounds **I** and **II** were obtained using single crystals grown by crystallization from ethanol. Table 1 shows the crystallographic and structure refinement data for the compounds **I** and **II**. The bond distances C(27)–O(1) and C(27)–O(2) of the carboxylate ion in **I** were 1.24(2) Å and 1.25(2) Å, respectively (Table 2, Figure 3). The bond distances indicated delocalisation of the electron density on the carboxylate group, with none of the two bonds being distinctly a single or double bond. The bond distance of C(28)–O(3) was 1.20(2) Å and was attributable to the C=O double bond whilst the bond distance of C(28)–O(4) was 1.32(2) Å indicating C–O single bond of the non-ionized carboxylic acid group. These bond distances were consistent with those reported in literature [18,19]. The bond length of N(2)–C(5) was 1.28(2) Å which was indicative of the C=N double bond whilst the bond length of N(1)–C(1) was 1.47(2) Å which confirmed the C–N single bond. The bond angle of C(2)–C(1)–C(4) was 109.1(1)° confirming the tetrahedral geometry (sp^3^) of the carbon C(3). The bond angle of C(6)–C(5)–C(4) was 121.2(1)° which was consistent with the trigonal planar geometry (sp^2^) of C5. The rigidity imposed by the C=N bond in the 7-membered ring forced it assume a distorted pseudo-chair conformation. The distorted conformation of **I** was further confirmed by the torsion angles which were C(12)–N(2)–C(5)–C(6) = 178.8(1)°, C(6)–C(5)–C(4)–C(1) = −108.2(1)°, C(2)–C(1)–N(1)–C(11) = 89.5(1)°, N(1)–C(11)–C(12)–N(2) = −0.71(2)°.

**Table 1:** Crystallographic data and structure refinement for compounds **I** and **II**.

Property	Compound I	Compound II
Formula	C_12_H_17_N_2_C_8_H_5_O_2_	C_12_H_16_N_2_
Formula Weight	354.40	188.27
Temperature (K)	200	200
Crystal System	triclinic	orthorhombic
Space group	*P*-1	*P*na 21
*a* (Å)	9.3608(4)	12.1454(3)
*b* (Å)	9.5706(3)	7.2730(2)
*c* (Å)	11.9881(4)	11.9222(3)
α (˚)	101.128(1)	90
β (˚)	102.728(1)	90
γ (˚)	114.297(1)	90
V (Å^3^)	904.91(6)	1053.13(5)
*Z*	2	4
*D* (calc) (g/cm^3^)	1.301	1.187
μ(MoKa) (mm)	0.091	0.091
F(000)	376	408
Crystal Size (mm)	0.15 × 0.36 × 0.42	0.19 × 0.44 × 0.45
Radiation (Å)	Mo Kα 0.71073	Mo Kα 0.71073
θ Min–Max (˚)	2.5–28.3	3.3–28.3
Data set	−12:12; −12:12, −15:15	−15:16; −9:9;−10:15
Tot. Uniq. Data R(int)	16298, 4492, 0.015	9541, 2371, 0.015
Observed data (I > 2.0 sigma (I))	3854	2285
*N*_ref_, *N*_par_	4492, 240	2371, 134
*R*, *Wr2*, *S*	0.0385, 0.1045, 1.04	0.0306, 0.0802, 1.03
Max and Av. Shift/Error	0.00, 0.00	0.00, 0.00
Min and Max, Resd Dens (e/Å^3^)	0.20, 0.30	−0.20, 0.18

**Table 2:** Selected bond lengths (Å), angles (°) and torsion angles for compounds **I** and **II**.

Property	I	II
**Bond length**		
C(27)–O(1)	1.24(2)	
C(27)–O(2)	1.25(2)	
C(28)–O(3)	1.20(2)	
C(28)–O(4)	1.32(2)	
N(2)–C(5)	1.28(2)	1.28(2)
N(1)–C(1)	1.47(2)	1.48(2)
**Bond angles**		
C(2)–C(1)–C(4)	109.1(1)	108.6(1)
C(4)–C(5)–C(6)	121.2(2)	117.5(1)
C(13)–C(12)–N(2)	117.7(2)	116.9(1)
C(16)–C(11)–N(1)	121.2(1)	119.7(1)
**Torsion angles**		
C(12)–N(2)–C(5)–C(6)	178.8(1)	178.1(1)
C(6)–C(5)–C(4)–C(1)	−108.2(1)	−107.0(2)
C(2)–C(1)–N(1)–C(11)	89.5(1)	94.5(2)
N(1)-C(11)–C(12)–N(2)	−0.7(2)	−2.9(2)

**Figure 4:**
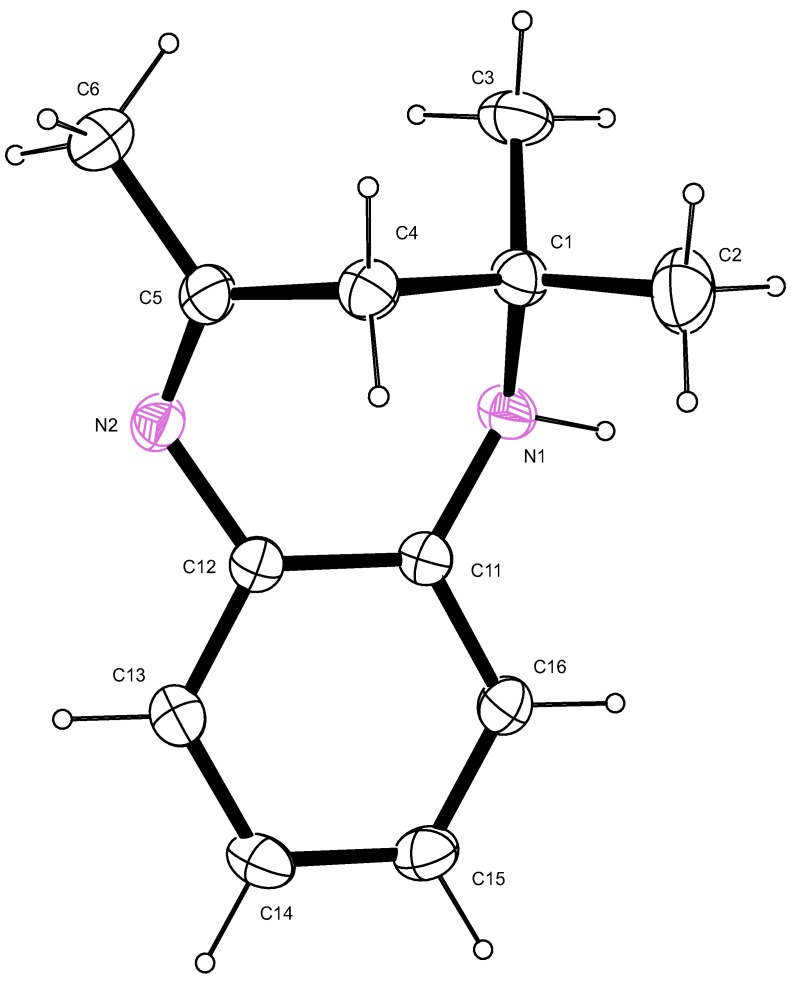
An ORTEP view of II showing 50% probability displacement ellipsoids and the atom labelling.

Similarly, for compound **II**, the bond length of the N(1)-C(1) single bond was 1.4802(2) Å whilst that of the N(2)–C(5) double bond was 1.282(2) Å (Table 2, Figure 4). The bond angle of the sp^2^ carbon was N(2)–C(5)–C(4) = 123.7(1)° confirming that the geometry of carbon C(5) is trigonal planar. The sp^3^ carbon C(1) had a bond angle of N(1)–C(1)–C(4) = 108.6(1)° which is consistent with its tetrahedral geometry. The torsion angles also confirmed the lack of planarity of the seven membered ring (Table 2).

### 2.3. Proposed Reaction Mechanism

**Scheme 2:**
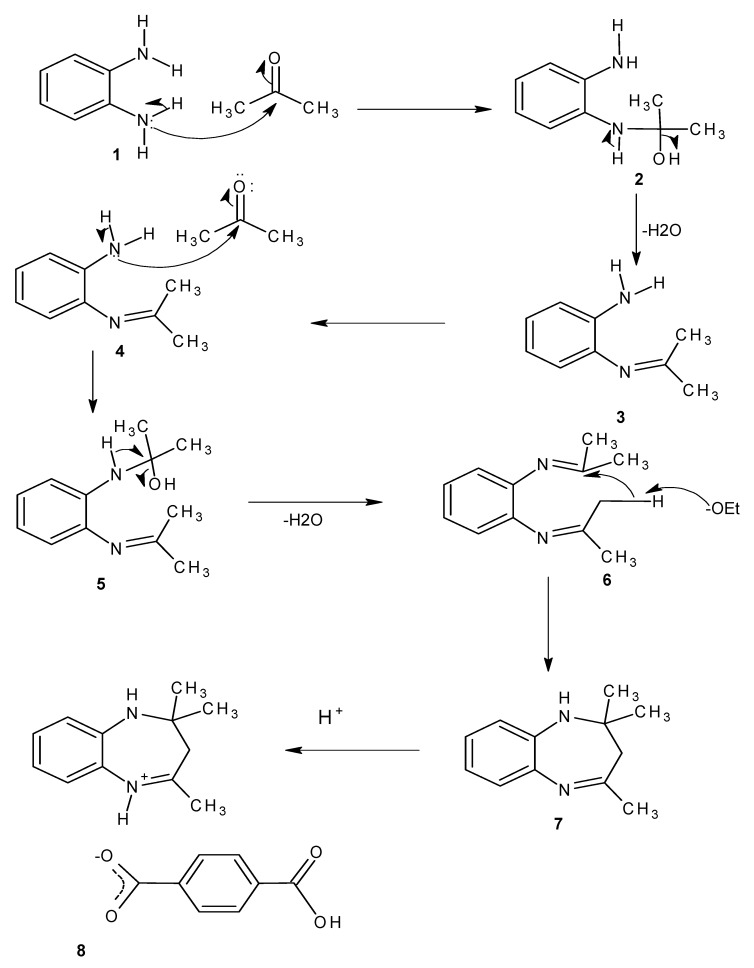
A proposed mechanism for the formation of compounds **I** and **II**.

Scheme 2 shows the proposed reaction mechanism for the formation of the benzodiazepine. It is proposed that the initial step is the attack of the carbonyl carbon of acetone by the lone pair of electrons on the amino group. Due to the difference in electronegativity between the carbon atom and the oxygen atom of the carbonyl group, the electron density is shifted slightly more towards the oxygen than the carbon, making the oxygen acquire a partial negative charge and the carbon atom a partial positive charge. Also, the tendency of the nitrogen to attract electrons towards itself making the hydrogen (N–H) easily abstracted, thereby leaves the nitrogen with a negative charge, making it a better nucleophile to attack the carbonyl in **1**. Loss of a water molecule from **2** results in the formation of a C=N bond in **3**. The second amine group attacks the carbonyl of another acetone molecule in **4** resulting in the formation of **5**, and the subsequent loss of a water molecule leads to the formation of the C=N group in **6** [20]. The ethoxide ion, formed from the dissociation of ethanol, abstracts a proton from the methyl group, resulting in the formation of the enolate ion in **6**. Since ethanol is a weak acid, it produces a strong conjugate base that can easily deprotonate a weakly acidic proton, in this case from a methyl group which is made acidic by the presence of unsaturation and a heteroatom on the adjoining carbon [12,21]. The loss of the proton by the methyl group makes it a good nucleophile which then attacks the carbon of the C=N bond because of the partial positive charge of the carbon as a result of the electron withdrawing effect of the nitrogen forming the benzodiazepine (**7**). In the case of compound one the benzodiazepine formed in **7** is then protonated by the isophthalic acid to form an iminium ion which subsequently forms a salt with the isophthalate ion in **8**.

## 3. Experimental

### 3.1. Reagents and Instrumentation

All the reagents were obtained from Sigma-Aldrich (St. Louis, MO, USA) and used without further purification. Melting points were determined on the Electrothermal 9100 melting point apparatus and were uncorrected. Thermal analysis experiments were performed on a TA Instruments DSC QA 100.Microwave experiments were performed in a CEM Discover and Explorer Benchmate. FT–IR spectra were recorded in a Bruker platinum ATR spectrophotometer Tensor 27. NMR spectra (DMSO) were recorded using a Bruker Avance AV 400 MHz spectrometer operating at 400 MHz for ^1^H and 100 MHz for ^13^C. Elemental analyses were performed using a Vario Elementar Microcube ELIII. Chromatographic analysis was performed using a Hewlett-Packard 6890 gas chromatograph coupled with a Hewlett-Packard 5973 mass spectrometer at 70 eV.

### 3.2. Synthesis of Benzodiazepium Isophthalate Salt and Benzodiazepine

#### 2,2,4-Trimethyl-2,3-dihydro-1H-1,5-benzodiazepin-5-ium isophthalate (**I**)

*o*-Phenylenediamine (0.01 mol) and isophthalic acid (0.01 mol) were subjected to microwave irradiation at 180 W (50 °C) for 15 min, after which an ethanol-acetone mixture (2 mL, 3:1) was added to dissolve the solidified reaction mixture. The reaction mixture was allowed to stand for 12 h during which a solid product was formed. Finally, the solid was filtered and the product (84%) was obtained as a yellow solid after recrystallization from ethanol. Mp 168–172 °C (DSC melting range: 166.3–181.3 °C), δ_H_: 8.48 (s, 1H, C22-phthalate), 8.16 (d, *J* = 7.7 Hz, 2H, C24– and C26– phthalate) 7.64 (t, *J* = 7.8 Hz, 1H, C25- phthalate), 6.84–6.95 (m, 2H, C14– and C15–H), 6.79 (d, *J* = 7.5 Hz, 2H, C13– and C16– H), 3.50–4.50 (br s, 2H, N–H), 2.51 (s, 3H. C6–Me), 2.16 (s, 2H, C4–CH_2_), 1.23 (s, 6H, C2– and C3–Me). δ_C_: 171.00 (C=N), 166.68 (C=O), 139.50 and 139.10 (C11 and C12), 133.49 (C24 and C26, phthalate), 131.35 (C23 and C21, phthalate), 130.07 (C22, phthalate), 129.27 (C25, phthalate), 126.89 and 125.29 (C14 and C15), 121.21 and 120.06 (C13 and C16), 66.45 (C1), 45.24 (C4), 29.95 (C2– and C3–Me), 29.32 (C6–Me). IR (ν_max_, cm^−1^): 1,710 (C=O), 1,607 (C=N), 1,208 (C–N), 1,552 (COO^−^), 3,309 (N−H), HRMS: *m/z* 189.1392 [M_A_^+^ = 189], *m/z* 165.0195^−^ [M_B_^−^ = 165]. Anal. calcd. for C_12_H_22_N_2_O_4_: C, 67.79; H, 6.21; N, 7.90. Found: C, 67.80; H, 6.24 N, 7.49.

#### 2,2,4-Trimethyl-2,3-dihydro-1H-1,5-benzodiazepine (**II**)

*o*-Phenylenediamine (0.02 mol) was heated under reflux with ethanol (10 mL) and acetone (5 mL) at 80 °C for 8 h. The solvent was removed under vacuum to give a light brown oily residue which was then redissolved in ethanol and placed in the refrigerator for 48 h. The product (62%) was obtained as a yellow solid after recrystallization from ethanol. M.p. 124–125 °C. ^1^H-NMR: δ_H_: 6.89–6.93 (m, 2H, C14–and C15–H), 6.79–6.87 (m, 2H, C13–and C16–H), 4.71 (s, N–H), 2.22 (s, 3H, C6–Me), 2.16 (s, 2H, C4), 1.24 (s, 6H, C2– and C3–Me). δ_C_: 170.74 (C=N), 139.34 and 139.10 (C11 and C12), 126.84 and 125,02 (C14 and C15), 121.02 and 119.86 (C13 and C16) 66.54 (C1), 45.24 (C4), 29.97 (C2– and C3–Me), 29.37 (C6–Me). IR: (ν_max_, cm^−1^) 3,294.25 (N–H), 2,964.39 (aliphatic C), 1,633.21(C=N), 1,430.09 (C–N). Anal calcd. for C_12_H_22_N_2_: C,76.60; H, 8.51; N,14.89. Found: C, 76.17; H, 8.47; N, 14.76.

### 3.3. X-ray Crystallography

X-ray diffraction analyses of **I** and **II** were performed at 200 K using a Bruker Kappa Apex II diffractometer with graphite monochromated Mo Kα radiation (*λ* = 0.71073 Å). APEXII [22] was used for data collection and SAINT [22] for cell refinement and data reduction. The structure was solved by direct methods using SHELXS–2013 [23] and refined by least-squares procedures using SHELXL-2013 [23] with SHELXLE [24] as a graphical interface. All non-hydrogen atoms were refined anisotropically. Carbon-bound H atoms were placed in calculated positions (C–H 0.95 Å for aromatic carbon atoms and C–H 0.99 Å for methylene groups) and were included in the refinement in the riding model approximation, with *U*_iso_(H) set to 1.2*U*_eq_(C). The H atoms of the methyl groups were allowed to rotate with a fixed angle around the C–C bond to best fit the experimental electron density (HFIX 137 in the SHELX program suite [23]), with *U*_iso_(H) set to 1.5*U*_eq_(C). The H atom of the hydroxyl group was allowed to rotate with a fixed angle around the C–O bond to best fit the experimental electron density (HFIX 147 in the SHELX program suite [23]), with *U*_iso_(H) set to 1.5*U*_eq_(O). Nitrogen-bound H atoms were located on a difference Fourier map and refined freely. Data were corrected for absorption effects using the numerical method implemented in SADABS [22]. CCDC 960105 and CCDC 960106 contains the supplementary crystallographic data for this paper. These data can be obtained free of charge via http://www.ccdc.cam.ac.uk/conts/retrieving.html (or from the CCDC, 12 Union Road, Cambridge CB2 1EZ, UK; Fax: +44 1223 336033; E-mail: deposit@ccdc.cam.ac.uk).

## 4. Conclusions

An unexpected 2,2,4-trimethyl-2,3-dihydro*-1H-*1,5-benzodiazepin-5-ium cation was synthesized by the reaction of phenylenediamine and isophthalic acid in the presence of acetone and ethanol under microwave irradiation without the presence of a catalyst, and it crystallized with the isophthalate anion. The formation of the 7-membered ring, without the involvement of isophthalic acid, was also confirmed by performing the reaction under conventional heating conditions by a reaction of *o*-phenylenediamine with acetone in ethanol. The synthesis method outlined here could be useful in the synthesis of derivatives of the seven membered benzodiazepine ring.

## Supplementary Materials

Supplementary materials can be accessed at: http://www.mdpi.com/1420-3049/18/11/14293/s1.
