# Supplementary material for: The Formation of 2,2,4-Trimethyl-2,3-dihydro-1H-1,5-Benzodiazepine from 1,2-Diaminobenzene in the Presence of Acetone
Source: Molecules. 2013 Nov 19;18(11):14293–305. doi: 10.3390/molecules181114293 (PMC6269685; doi:10.3390/molecules181114293)

Supplementary Materials

Figure S1. IR spectrum of compound I.

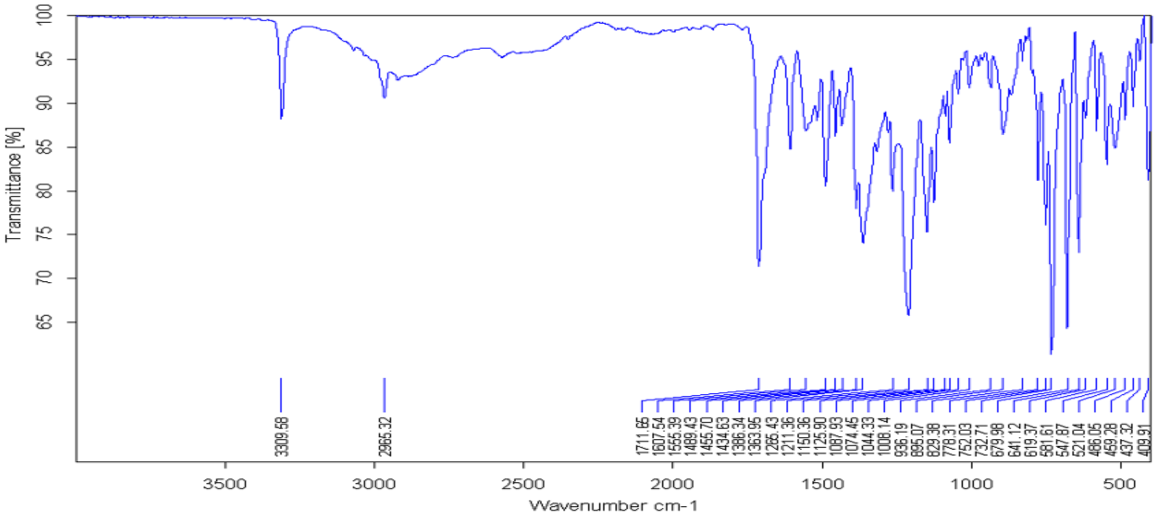

Figure S2. IR spectrum of compound II.

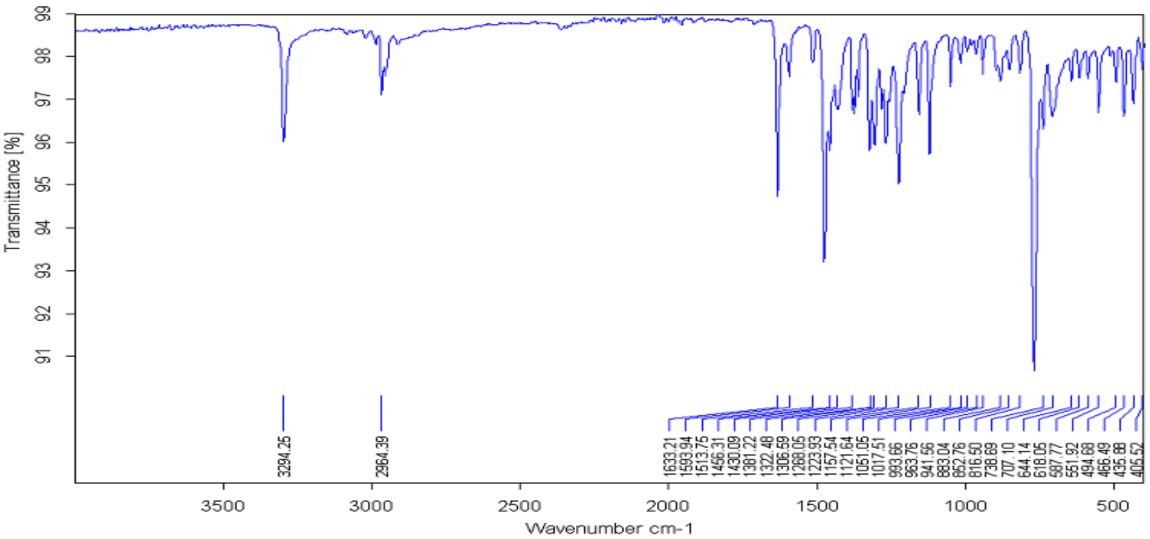

Figure S3. DEPT spectrum of compound I.

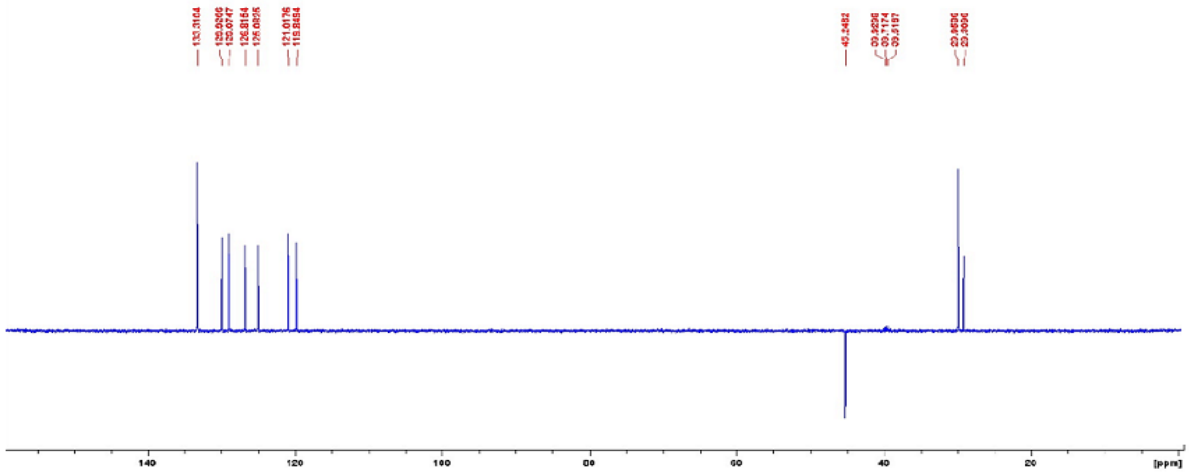

Figure S4. Mass spectrum of isophthalate ion.

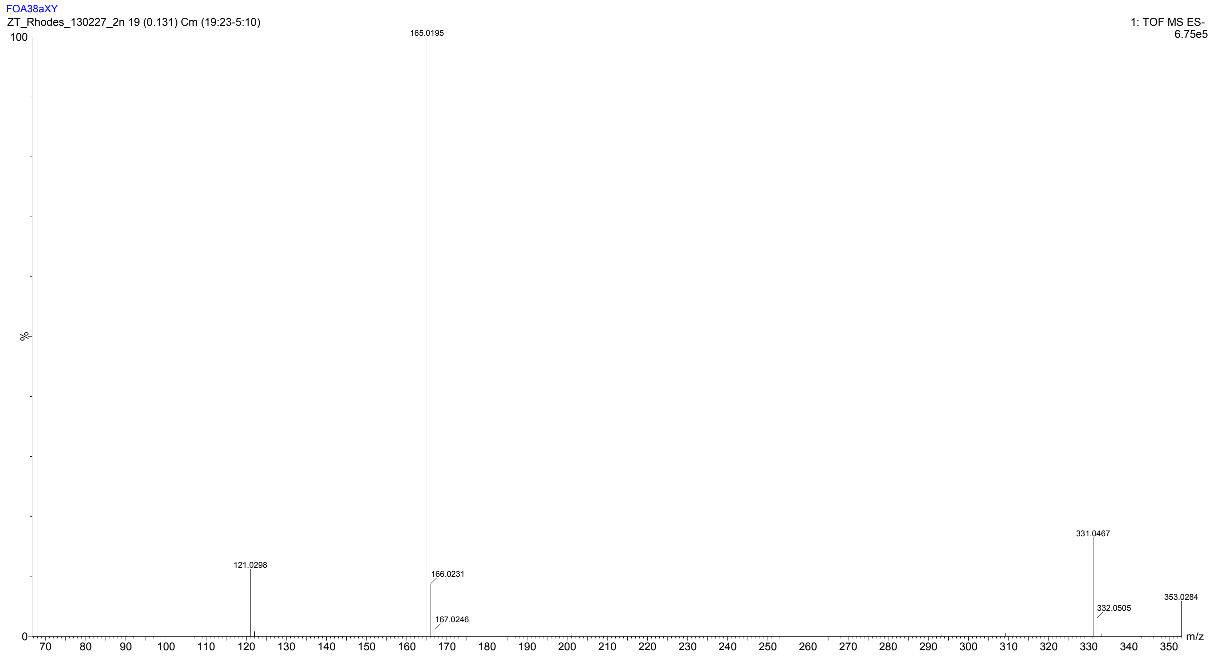

Figure S5. Mass spectrum of benzodiazepinium ion.

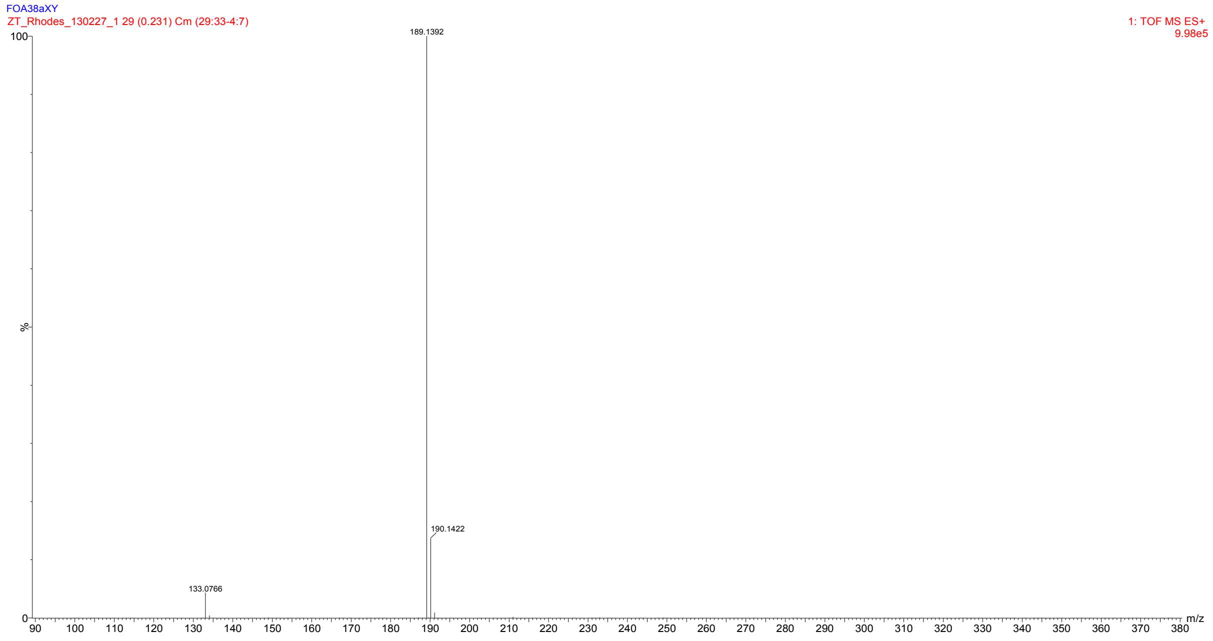

Figure S6. ^{1}H-NMR spectrum of compound II.

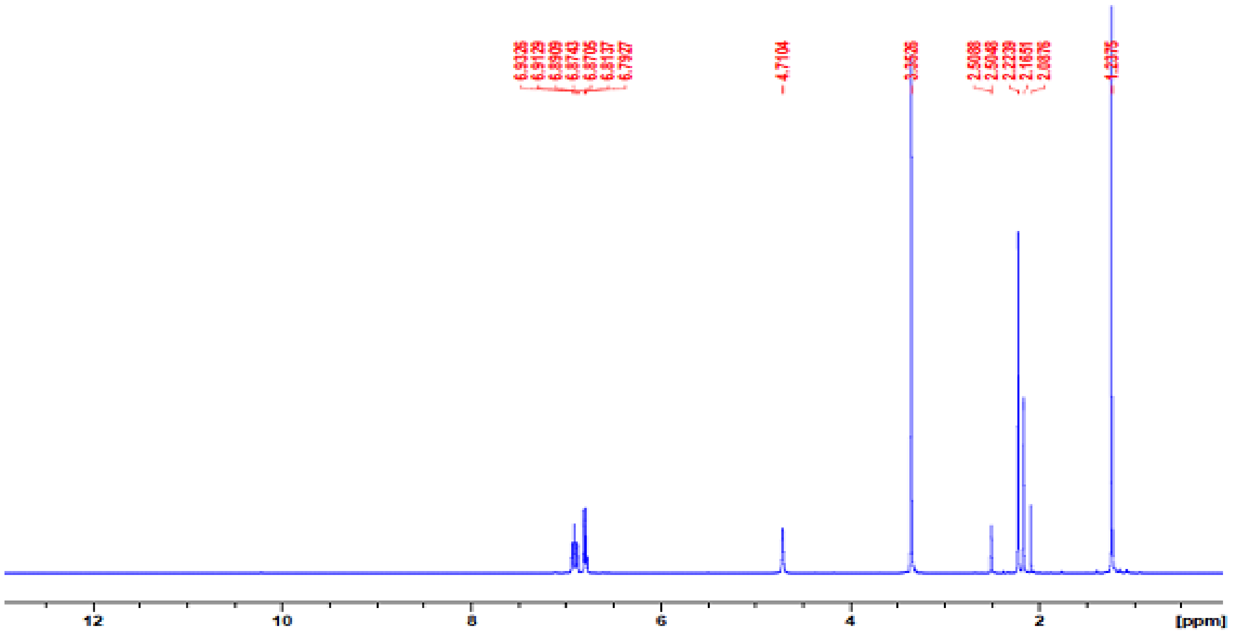

Figure S7. ^{13}C-NMR spectrum of compound II.

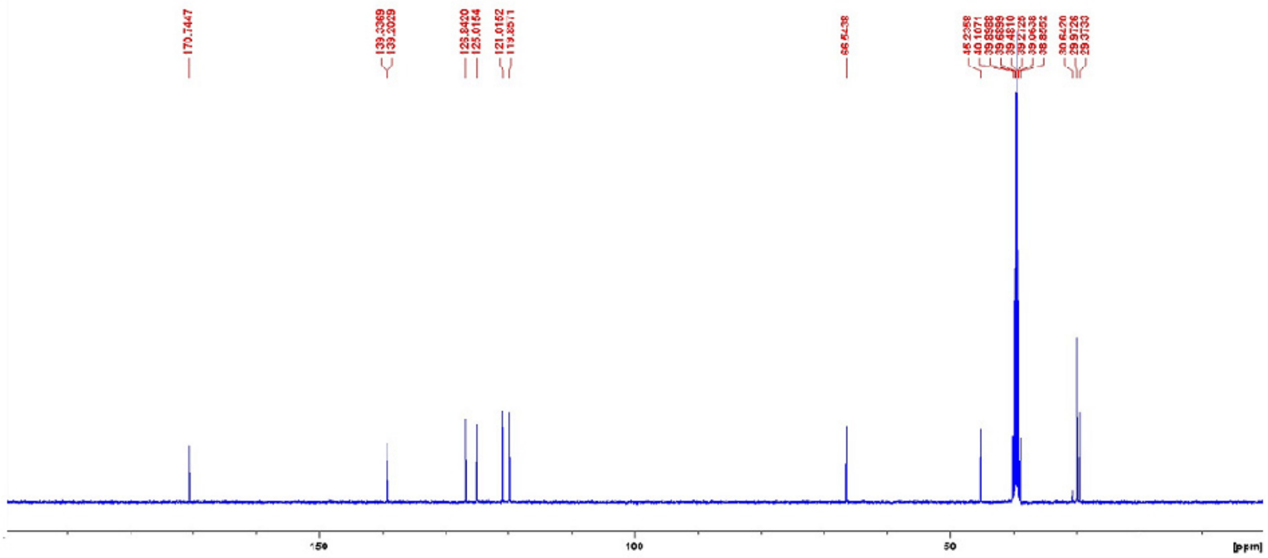

Figure S8. DEPT spectrum of compound II.

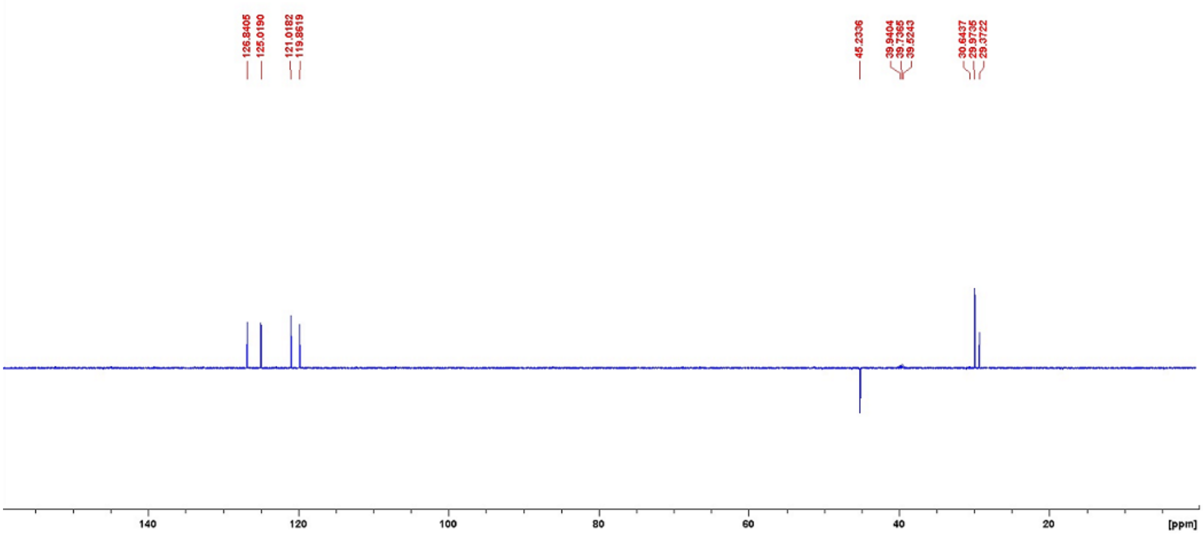

Supplement: Supplementary file 1 [file molecules-18-14293-s001.pdf]
